# COVID-19 in discharged patients with diabetes and chronic kidney disease: one-year follow-up and evaluation

**DOI:** 10.3389/fendo.2025.1519993

**Published:** 2025-02-04

**Authors:** Enrong Ran, Yutong Zou, Chuanyi Zhao, Kai Liu, Jiamin Yuan, Wenjie Yang, Lijun Zhao, Qing Yang, Jia Yang, Xuegui Ju, Linli Cai, Yanlin Lang, Xingyuan Li, Ke Liu, Fang Liu

**Affiliations:** ^1^ Department of Nephrology, West China Hospital, Sichuan University; Laboratory of Diabetic Kidney Disease, Kidney Research Institute, West China Hospital, Sichuan University, Chengdu, Sichuan, China; ^2^ Department of Nephrology, Suining Central Hospital, Suining, China; ^3^ Department of Clinical Research Management, West China Hospital of Sichuan University, Chengdu, China; ^4^ Division of Project Design and Statistics, West China Hospital of Sichuan University, Chengdu, China

**Keywords:** COVID-19, diabetes, chronic kidney disease, acute kidney injury, mortality

## Abstract

**Purpose:**

To evaluate the all-cause mortality rate and renal outcomes in patients with diabetes and chronic kidney disease (CKD) following hospital discharge for COVID-19.

**Methods:**

This single-center prospective observational study included 187 discharged COVID-19 patients with diabetes and CKD, admitted between December 2022 and January 2023 at West China Hospital, Sichuan University. Cox regression analysis was used to assess mortality risk, and logistic regression was applied to identify risk factors for rapid CKD progression after discharge.

**Results:**

During the one-year follow-up, the all-cause mortality rate was 26.7%, with a COVID-19-related acute kidney injury (AKI) incidence of 35.3%, and 35.8% of patients experienced rapid CKD progression after discharge. Cox proportional hazards regression indicated that sepsis and mechanical ventilation were major risk factors for post-discharge all-cause mortality. Logistic regression identified baseline eGFR < 60 mL/min/1.73 m² as an independent risk factor for rapid CKD progression.

**Conclusions:**

During the one-year follow-up period, we observed that patients with diabetes and CKD exhibited higher all-cause mortality and experienced rapid deterioration of kidney function after acute infection with COVID-19. This underscores the importance of ongoing longitudinal follow-up to more accurately track the long-term health effects of COVID-19 on patients with diabetes and CKD.

## Introduction

Due to immune dysfunction, patients with underlying conditions are at increased risk of serious complications and higher mortality when exposed to the novel coronavirus disease (COVID-19) (1–3). People with diabetes are twice as likely to develop COVID-19 pneumonia and have higher in-hospital mortality compared to people without diabetes (4, 5). In patients with chronic kidney disease (CKD), COVID-19 infection can significantly worsen kidney function and lead to complications such as acute kidney injury (AKI), which is common in critically ill COVID-19 patients and is associated with higher mortality rates (6). Patients with diabetes and CKD face even more complex health challenges when infected with COVID-19, which significantly increases disease severity and associated mortality. Evaluating the long-term impact of COVID-19 on this vulnerable population may help to develop targeted management strategies.

In December 2022, China experienced the peak of the COVID-19 epidemic, leading to a surge in hospitalizations for COVID-19 within a short period. Our previous study reported that among hospitalized patients with diabetes and CKD, the incidence of acute kidney injury (AKI) was 21%, sepsis was 4.7%, respiratory failure was 40%, the proportion receiving mechanical ventilation was 30.1%, and the initial hospital mortality was 26.3% (7). Despite our greater understanding of the clinical characteristics of hospitalized patients (8), we still know very little about their long-term survival and renal outcomes after discharge. Current follow-up studies have focused on symptoms and lung assessment (9, 10), and little is known about the progression of CKD after COVID-19 infection. In addition, the heterogeneity of COVID-19 follow-up studies has led to a lack of specific data on long-term mortality, which limits our in-depth understanding of long-term health outcomes for discharged patients. Therefore, we initiated a prospective observational study to assess the risk of death and changes in kidney function in patients within one year of discharge from the hospital, in order to better understand the long-term impact of COVID-19 and improve patient management.

## Methods

### Patients

This was a single-center prospective observational cohort study. Data were collected from the inpatient electronic medical record system of West China Hospital, Sichuan University. A total of 338 patients with diabetes (including both type 1 and type 2 diabetes) and CKD combined with COVID-19 infection between December 7, 2022, and January 31, 2023, were included. Patients who died during hospitalization or had no visit history were excluded, as well as patients with end-stage renal disease (ESRD) who had already begun renal replacement therapy before COVID-19. Discharged patients were followed up in outpatient clinics or by telephone. The primary outcome was all-cause mortality after discharge, and the secondary outcomes included the proportion of patients with rapid progression of CKD and COVID-19-associated AKI.

### Definitions

AKI is defined as an increase in serum creatinine (Scr) of 0.3 mg/dL within 48 hours, or 50% over 7 days compared to baseline, according to KDIGO criteria. If there was a downward trend in Scr post-discharge, the baseline was recalculated using the value of Scr after discharge. Estimated glomerular filtration rate (eGFR) was calculated using the CKD Epidemiology Collaboration (CKD-EPI) creatinine equation. In patients with AKI, failure to recover renal function means a decrease in Scr of less than 50% of its peak or the need to continue hemodialysis. CKD staging was based on eGFR levels: Stage 1 (≥ 90 mL/min/1.73 m²), Stage 2 (60-89 mL/min/1.73 m²), Stage 3 (30-59 mL/min/1.73 m²), Stage 4 (15-29 mL/min/1.73 m²), and Stage 5 (< 15 mL/min/1.73 m² or dialysis). CKD rapid progression was defined as a decrease in eGFR of at least 5 mL/min/1.73 m² during the follow-up period (11). Mechanical ventilation during hospitalization included invasive mechanical ventilation and non-invasive mechanical ventilation.

The severity of COVID-19 infections is categorized according to the Chinese Clinical Guidance for COVID-19 Infection Diagnosis and Treatment (7th edition), published by the Chinese National Health Commission. The moderate type is characterized by fever, respiratory symptoms, and imaging findings indicative of pneumonia. Severe infections are marked by shortness of breath, low resting oxygen saturation, a PaO2/FiO2 ratio of ≤300 mmHg, a standard respiratory rate of ≥30 breaths per minute, and a resting oxygen saturation of ≤93%. Critically severe cases are identified by respiratory failure necessitating mechanical ventilation, shock, or the failure of other organs requiring intensive care.

### Covariates

Demographic data were collected during hospitalization, including concomitant diseases, severity of COVID-19, major complications, medications, and more. Hypertension and other comorbidities were identified based on ICD-10 codes. Scr levels were recorded at the time of discharge and again at 6 to 12 months post-discharge.

### Statistical analysis

Continuous variables with a normal distribution were expressed as mean ± standard deviation and compared using the t-test; categorical variables were expressed as percentages and compared using the χ² test or Fisher’s exact test. Univariate and multivariate Cox regression analyses were used to evaluate risk factors for post-discharge mortality. In the construction of the Cox regression model, we used stepwise AIC-based selection, combining forward selection and backward elimination, to identify significant predictors while including clinically relevant variables. The proportional hazards assumption of the model was tested using Schoenfeld residuals, and time-dependent interaction analyses were performed for variables that might violate the proportional hazards assumption to assess their impact on the model.

Because some patients had inconsistent renal function follow-up times, making it difficult to determine the exact time of CKD progression, we chose to use univariate and multivariate logistic regression analyses to evaluate risk factors for rapid CKD progression. For missing covariates, multiple imputation was used to impute missing data. P values <0.05 are considered statistically significant. Statistical analysis was performed using RStudio software, and figures were generated using GraphPad Prism 10.

## Result

### Clinical characteristics

**Figure 1:**
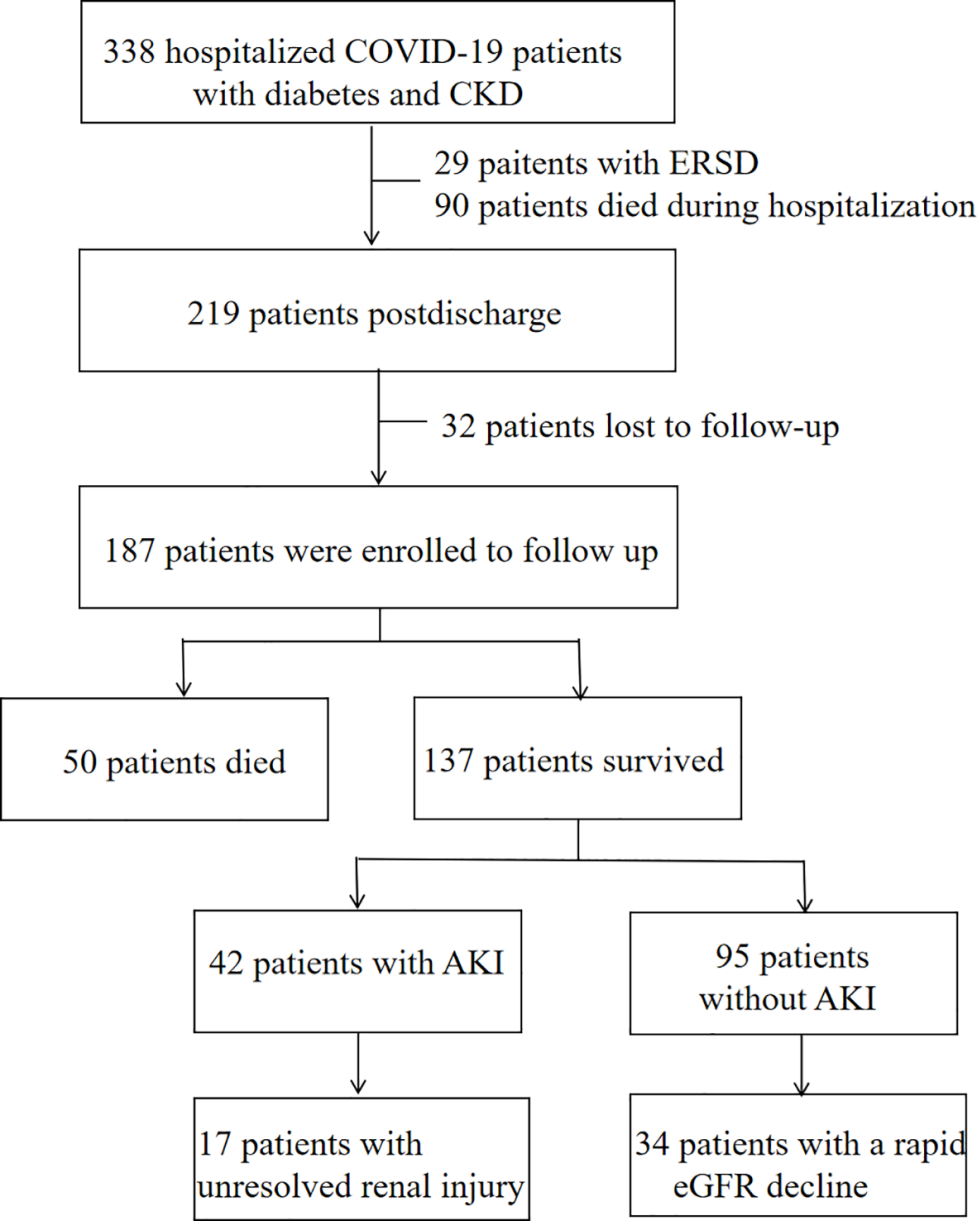
Flow chart of hospital survivors with COVID-19.

Between December 7, 2022, and January 31, 2023, a total of 338 patients diagnosed with COVID-19 who had diabetes and CKD were admitted. Of these patients, 29 patients with end-stage renal disease were excluded, 90 died during the initial period of hospitalization, and 219 were discharged. Discharged patients were followed up until December 2023. During the follow-up period, 32 patients were lost to follow-up. The final study cohort consisted of 187 patients (**Figure 1**).

The mean age of patients in the study cohort was 68.7 ± 15.3 years, and 63.6% were male. The majority of patients (48.7%) were diagnosed with moderate COVID-19, with 20.9% and 30.5% of patients diagnosed with severe and critical infections, respectively. The distribution of CKD stages was as follows: 16.0% of patients were in CKD stage 1-2, 66.3% were in CKD stage 3-4, and 17.6% were in CKD stage 5. Compared with CKD stage 1-2 patients, CKD stage 3-5 patients were significantly older, had a lower proportion of severe COVID-19, higher NT-proBNP and uric acid, and lower hemoglobin and bilirubin (**Table 1**).

**Table 1:** Baseline characteristics of discharged patients during hospitalization.

Variables	Overall (n=187)	CKD stage 1-2 (n=30)	CKD stage 3-4 (n=124)	CKD stage5 (n=33)	P-value
Male(n,%)	128 (68.4)	18 (60)	87 (70.2)	23 (69.7)	0.553
Age, years	68.7 ± 15.3	63.1 ± 15.5	70.8 ± 14.6	65.6 ± 16.5	0.020
Alcohol(n,%)	30 (16.3)	5 (17.2)	22 (17.9)	3 (9.4)	0.544
Smoking(n,%)	45 (24.3)	5 (17.2)	34 (27.6)	6 (18.2)	0.332
BMI, kg/m^2^	24.2 ± 4.0	24.7 ± 5.5	24.3 ± 3.5	23.2 ± 3.7	0.429
SBP, mmHg	134.7 ± 22.3	136.3 ± 20.2	133.3 ± 21.6	138.3 ± 26.7	0.475
DBP, mmHg	77.2 ± 14.4	79.5 ± 13.1	77.1 ± 14.4	75.3 ± 15.7	0.513
Diabetic duration, years	11.1 ± 7.6	8.9 ± 6.3	11.8 ± 8.0	10.0 ± 6.7	0.274
Hypertension(n,%)	157 (84.0)	23 (76.7)	106 (85.5)	28 (84.8)	0.470
Cardiovascular disease(n,%)	99 (52.9)	14 (46.7)	69 (55.6)	16 (48.5)	0.577
COVID-19 severity					0.016
Moderate(n,%)	91 (48.7)	11 (36.7)	61 (49.2)	19 (57.6)	
Severe(n,%)	39 (20.9)	3 (10)	32 (25.8)	4 (12.1)	
Critically severe(n,%)	57 (30.5)	16 (53.3)	31 (25)	10 (30.3)	
Laboratory results within hospital admission					
Lymphocyte count,×10^9/L	8.3 ± 4.8	8.8 ± 4.9	7.9 ± 4.5	9.2 ± 5.9	0.287
Hemoglobin, g/L	105.8 ± 27.7	112.9 ± 24.8	109.0 ± 26.0	87.2 ± 29.4	< 0.001
D-Dimer, mg/L	5.7 ± 8.2	6.8 ± 9.4	5.2 ± 8.5	6.3 ± 5.2	0.588
Albumin, g/L	33.0 ± 5.4	33.3 ± 6.2	33.6 ± 5.3	30.9 ± 5.0	0.039
Total bilirubin, umol/L	10.2 ± 7.6	12.8 ± 6.8	10.4 ± 7.7	6.7 ± 7.0	0.006
Total cholesterol, mmol/L	3.5 ± 1.2	3.4 ± 1.1	3.5 ± 1.2	3.6 ± 1.3	0.804
Triglyceride, mmol/L	2.0 ± 1.6	1.8 ± 1.3	2.0 ± 1.7	2.2 ± 1.2	0.626
Fasting glucose, mmol/L	11.1 ± 6.2	10.9 ± 3.9	11.0 ± 6.7	11.3 ± 6.1	0.970
NT-proBNP, ng/L	4297.7 ± 6212.5	2016.1 ± 3006.8	3949.5 ± 5976.9	8687.8 ± 8043.2	< 0.001
Scr, umol/L	228.0 ± 215.5	76.4 ± 18.6	165.6 ± 61.8	600.4 ± 274.6	< 0.001
eGFR, mL/min/1.73 m2	40.1 ± 25.7	85.9 ± 17.3	37.1 ± 12.2	8.9 ± 3.6	< 0.001
Uric acid, mmol/L	389.0 ± 214.8	258.1 ± 111.1	384.7 ± 220.5	524.5 ± 186.9	< 0.001

CKD, chronic kidney disease; BMI, Body mass index; SBP, Systolic blood pressure; DBP, Diastolic blood pressure; AKI, Acute kidney injury; NT-proBNP, N-terminal pro-B-type natriuretic peptide; Scr, Serum creatinine; eGFR, Estimated glomerular filtration rate.

P-values indicate the statistical difference in baseline characteristics between different CKD stages.

### Mortality

During the follow-up period, the all-cause mortality rate of discharged patients was 26.7% (50/187). Compared with patients who survived after discharge, patients who died had a higher proportion of critically severe COVID-19, respiratory failure, stroke, and AKI when hospitalized during the peak period of COVID-19, and the levels of D-dimer and NT-proBNP were significantly increased. In terms of treatment, patients who died received more immunoglobulin and diuretic therapy, but the use of Angiotensin-Converting Enzyme Inhibitors (ACEI)/Angiotensin II Receptor Blockers (ARB) was relatively less, and there was no significant difference between glucocorticoid and antiviral drug use. Among discharged patients who had experienced sepsis while hospitalized, 46% died after discharge, and among those who received mechanical ventilation, the mortality rate was as high as 63% (**Table 2**).

**Table 2:** Clinical characteristics during initial hospitalization of patients who died after discharge.

Variables	Total (n = 187)	Survival (n = 137)	Death (n = 50)	P-value
Male(n,%)	59 (31.6)	45 (32.8)	14 (28)	0.528
Age>70,years	97 (51.9)	69 (50.4)	28 (56.0)	0.495
Smoking(n,%)	45 (24.1)	35 (25.6)	10 (20.0)	0.404
BMI, kg/m^2^	24.2 ± 4.0	24.1 ± 3.6	24.6 ± 4.9	0.524
Hypertension(n,%)	157(83.9)	114 (83.2)	43 (86.0)	0.814
Cardiovascular disease(n,%)	99(52.9)	76 (55.5)	23 (46.0)	0.325
COVID-19 severity				< 0.001
Moderate(n,%)	91 (48.7)	77 (56.2)	14 (28)	
Severe(n,%)	39 (20.9)	35 (25.5)	4 (8)	
Critically severe(n,%)	57 (30.5)	25 (18.2)	32 (64)	
Complications during hospitalization				
Respiratory failure(n,%)	59 (31.6)	32 (23.4)	27 (54)	< 0.001
Sepsis(n,%)	13 (7.0)	7 (5.1)	6 (12.0)	0.113
Myocardial_infarction(n,%)	6 (3.2)	4 (2.9)	2 (4.0)	0.659
Stroke(n,%)	28 (15.0)	11 (8.0)	17 (34.0)	< 0.001
AKI(n,%)	66 (35.3)	42 (30.7)	24 (48.0)	0.028
Laboratory results within hospital admission				
Lymphocyte count,×10^9/L	8.3 ± 4.8	7.9 ± 4.3	9.4 ± 6.0	0.053
Hemoglobin, g/L	105.8 ± 27.7	107.3 ± 28.6	101.7 ± 25.0	0.223
D-Dimer, ng/mL	5.7 ± 8.2	3.8 ± 5.4	10.6 ± 11.6	< 0.001
Albumin, g/L	33.0 ± 5.4	33.2 ± 5.5	32.5 ± 5.2	0.383
Fasting glucose, mmol/L	11.1 ± 6.3	10.6 ± 5.2	12.2 ± 6.2	0.122
Total cholesterol, mmol/L	3.5 ± 1.2	3.6 ± 1.2	3.3 ± 1.3	0.137
Triglyceride, mmol/L	2.0 ± 1.5	2.0 ± 1.6	2.1 ± 1.5	0.623
NT-proBNP, pg/mL	4297.7 ± 6212.5	3654.3 ± 5364.8	5973.6 ± 7840.4	0.037
eGFR, mL/min/1.73 m2	40.1 ± 25.7	40.6 ± 26.1	38.8 ± 24.9	0.674
Treatment received during hospital				
Corticosteroids(n,%)	108 (57.8)	74 (54.0)	34 (68.0)	0.087
Antivirals (n,%)	52 (27.8)	36 (26.3)	16 (32.0)	0.440
Inflammatory factor inhibitors(n,%)	12 (6.4)	8 (5.8)	4 (8.0)	0.736
Intravenous immunoglobulin(n,%)	25 (13.4)	11 (8.0)	14 (28.0)	< 0.001
Diuretics(n,%)	148 (83.6)	103 (78.6)	45 (97.8)	0.002
Use of ACEI/ARB(n,%)	82 (46.3)	67 (51.1)	15 (32.6)	0.030
Use of SGLT2 (n,%)	26 (14.7)	23 (17.6)	3 (6.5)	0.069
Mechanical ventilation, n (%)	46 (24.6)	17 (12.4)	29 (58.0)	< 0.001
Hospital length of stay, days	13.4 ± 8.0	13.8 ± 7.9	12.4 ± 8.2	0.260

BMI, Body mass index; AKI, kidney injury; NT-proBNP, N-terminal pro-B-type natriuretic peptide; eGFR, Estimated glomerular filtration rate; ACEI, Angiotensin-converting enzyme inhibitors; ARB, Angiotensin receptor blockers; SGLT2, Sodium-glucose transport protein 2 inhibitors.

**Figure 2:**
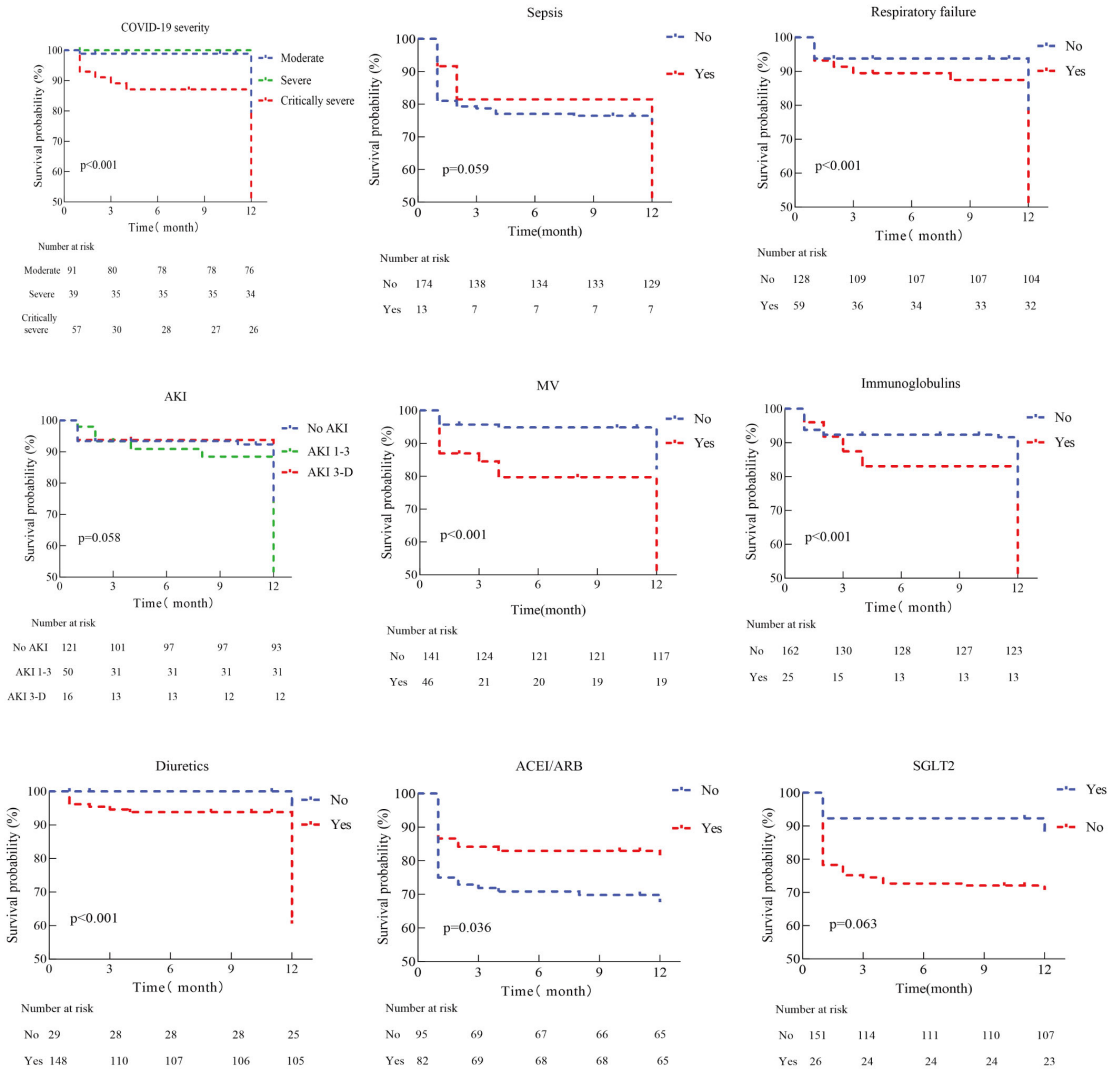
Kaplan-Meier analysis for death of discharge patients with diabetes and chronic kidney disease after COVID-19 infection. MV, Mechanical ventilation; AKI, Acute kidney injure; ACEI, Angiotensin-converting enzyme inhibitors; ARB, Angiotensin ii receptor blockers; SGLT2, Sodium-glucose transport protein 2 inhibitors.

Kaplan-Meier curves (**Figure 2**) show that patients with critically severe COVID-19, combined with respiratory failure, and treated with diuretics, immunoglobulin, or mechanical ventilation have a higher mortality rate, while patients with oral ACEI/ARB have a relatively low risk of death. Further analysis indicated that patients’ all-cause mortality peaked in the first three months after discharge. A Cox proportional hazards regression model was used to analyze the potential relationship between COVID-19 severity, complications, treatment received during hospitalization, and all-cause mortality after discharge. Univariate analysis revealed that critically severe COVID-19, respiratory failure, stroke, and AKI during the hospital course were significantly associated with post-discharge all-cause mortality. Patients who received mechanical ventilation, intravenous immunoglobulin, and diuretics had a higher risk of death. Additionally, high D-dimer and NT-proBNP levels were associated with an increased risk of death. In a multifactor analysis, we adjusted for possible confounding factors; sepsis (HR 2.76, 95% CI 1.06-7.17) and receiving mechanical ventilation (HR 3.88, 95% CI 1.73-8.68) were the main risk factors for all-cause mortality after discharge (**Table 3**).

**Table 3:** Risk factors of post-discharge mortality based on cox proportional hazard regression.

	Univariate		Multivariate	
	HR (95%CI)	P-value	HR (95%CI)	P-value
Sex, male	0.78 (0.42,1.45)	0.441	0.85(0.41,1.75)	0.665
Age,>70years	1.19 (0.68,2.07)	0.548	1.42(0.73,2.75)	0.297
Smoking	0.77 (0.38,1.53)	0.454	–	–
Hypertension	1.19(0.53, 2.64)	0.676	–	–
Cardiovascular disease	0.72 (0.41,1.26)	0.250	–	–
COVID-19 severity				
Moderate	–	–	–	–
Severe	0.65 (0.21,1.98)	0.449	0.54(0.17,1.73)	0.299
Critically severe	4.82 (2.57,9.05)	< 0.001^†^	2.32(0.81,6.65)	0.117
Hospital length of stay	0.97 (0.93,1.01)	0.185	0.97(0.94,1.01)	0.134
Respiratory failure	3.04 (1.74,5.31)	< 0.001^†^	1.26(0.57,2.77)	0.573
Sepsis	2.07 (0.88,4.86)	0.095	2.76(1.06,7.17)	0.037‡
Myocardial infarction	1.38 (0.34,5.7)	0.652	–	–
Stroke	3.70 (2.06,6.66)	< 0.001^†^	0.71(0.3,1.67)	0.436
AKI	1.91 (1.1~3.33)	0.022^†^	1.14(0.56,2.32)	0.715
Glucocorticoids	1.61 (0.89,2.92)	0.114	0.74(0.35,1.56)	0.436
Antivirals	1.29 (0.71,2.34)	0.398	–	–
Inflammatory factor inhibitors	1.40 (0.5,3.89)	0.518	–	–
Intravenous immunoglobulin	3.05 (1.64,5.66)	< 0.001^†^	2.06(0.98,4.35)	0.057
Diuretics	10.15 (1.4,73.64)	0.022^†^	–	–
Use of ACEI/ARB	0.51 (0.28~0.95)	0.034^†^	–	–
Use of SGLT2	0.36 (0.11~1.17)	0.090	–	–
Mechanical ventilation	6.17 (3.51,10.86)	< 0.001^†^	3.88(1.73,8.68)	0.001‡
Laboratory results within hospital admission				
Lymphocyte count,×10^9/L(per 1SD)	1.3 (1.03~1.66)	0.031^†^	0.97(0.91,1.03)	0.333
Hemoglobin, g/L(per 1SD)	0.85 (0.64~1.12)	0.253	–	–
D-Dimer, ng/mL(per 1SD)	1.69 (1.38~2.08)	<0.001^†^	1.01(0.98,1.05)	0.504
Albumin, g/L(per 1SD)	0.88 (0.67~1.17)	0.389	–	–
Total cholesterol, mmol/L(per 1SD)	0.79 (0.59~1.07)	0.134	–	–
Triglyceride, mmol/L(per 1SD)	1.06 (0.83~1.35)	0.632	–	–
Fasting glucose, mmol/L(per 1SD)	1.20 (0.95~1.52)	0.120	–	–
NT-proBNP, pg/mL(per 1SD)	1.29 (1.03~1.62)	0.028^†^	–	–
eGFR, mL/min/1.73 m2(per 1SD)	1.11 (0.86~1.44)	0.421	0.99(0.98,1.01)	0.372

HR, Hazard Ratio; CI, Confidence Interval; AKI, Acute Kidney Injury; ACEI, Angiotensin-Converting Enzyme Inhibitors; ARB, Angiotensin II Receptor Blockers; SGLT2, Sodium-Glucose Transport Protein 2 Inhibitors; NT-proBNP, N-Terminal Pro-B-Type Natriuretic Peptide; eGFR, Estimated Glomerular Filtration Rate.

The multivariate Cox regression model was adjusted for age, sex, COVID-19 severity, hospital length of stay, respiratory failure, sepsis, stroke, AKI, mechanical ventilation, use of glucocorticoids, and intravenous immunoglobulin, as well as laboratory variables including lymphocyte count, D-dimer, and eGFR.

†: Univariate COX analysis, P < 0.05; ‡: Multivariate COX analysis, P < 0.05.

### CKD progression

**Figure 3:**
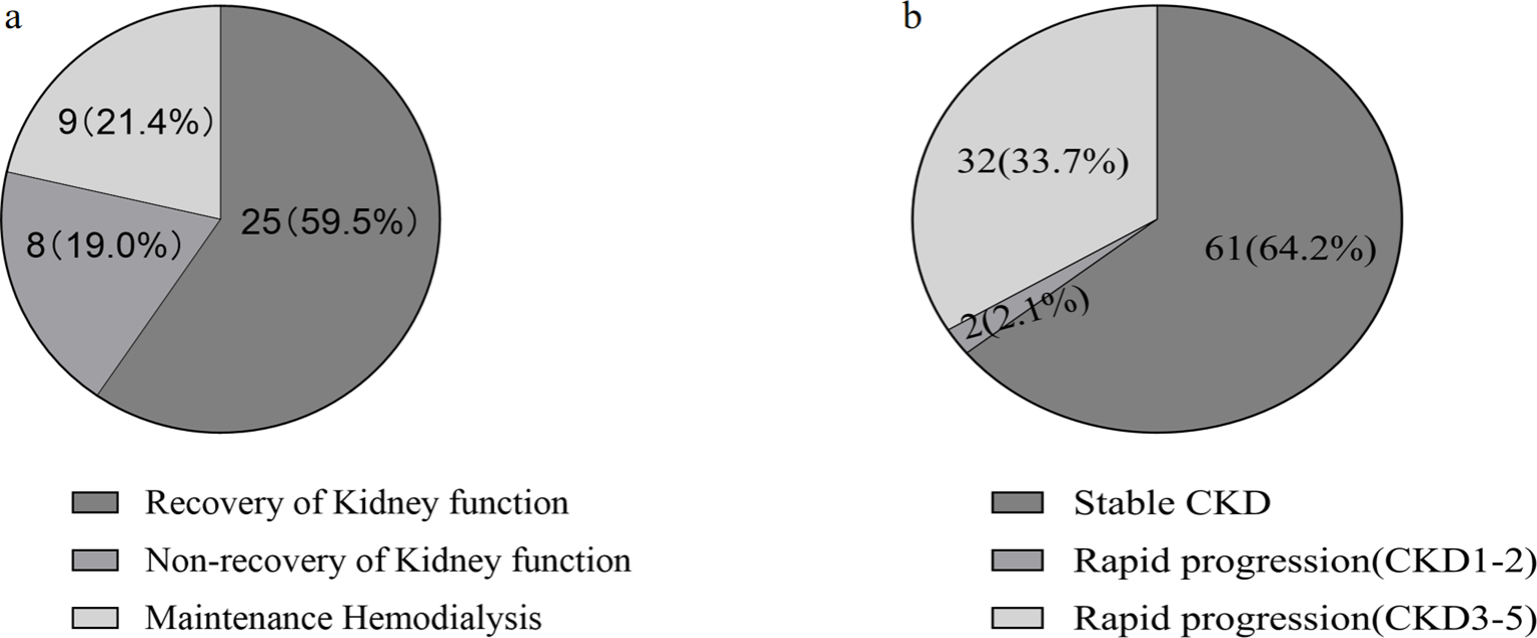
Analysis of renal outcomes in discharged COVID-19 patients with diabetes and chronic kidney disease (CKD) during one-year follow-up. **(A)** Patients developed aute kidney injure(AKI) during hospitalization. **(B)** Patients without AKI during hospitalization.

Of the patients who survived after discharge, 95 did not develop AKI during hospitalization. However, during follow-up, 35.8% of them developed rapid progression of CKD (**Figure 3**). Patients with CKD progression had lower baseline eGFR levels compared to patients without CKD progression. There were no significant differences in gender, age, BMI, or severity of COVID-19 infection between the two groups. In addition, HbA1c levels decreased during follow-up compared to the acute phase of COVID-19 infection. Patients with CKD progression tended to have lower HbA1c levels (**Table 4**). Logistic regression univariate analysis (OR 3.69, 95% CI 1.27-10.71) and multivariate analysis (OR 7.14, 95% CI 1.13-45.15) showed that a baseline eGFR < 60 mL/min/1.73 m² was an independent risk factor for renal function progression (**Table 5**).

**Table 4:** Comparative analysis of Scr, eGFR, and Hb1Ac during follow-up: rapid progression of renal function vs. no progression.

Variables	Total (n=95)	No Progression (n=61)	Progression (n=34)	P-value
Sex(male)	59 (62.1)	35 (57.4)	24 (70.6)	0.203
Age, years	68.9 ± 16.1	70.2 ± 16.3	66.5 ± 15.6	0.285
BMI, kg/m^2^	23.8 ± 3.3	23.8 ± 3.5	23.6 ± 3.1	0.793
Diabetic duration, years	10.6 ± 7.4	11.3 ± 7.8	9.4 ± 6.8	0.359
Hypertension((n,%))	80 (84.2)	52 (85.2)	28 (82.4)	0.711
Cardiovascular disease(n,%)	53 (55.8)	32 (52.5)	21 (61.8)	0.381
COVID-19 severity				0.101
Moderate(n,%)	51 (53.7)	28 (45.9)	23 (67.6)	
Severe(n,%)	28 (29.5)	20 (32.8)	8 (23.5)	
Critically severe(n,%)	16 (16.8)	13 (21.3)	3 (8.8)	
CKD3-5(n,%)	77 (81.1)	54 (88.5)	23 (67.6)	0.013
Admission				
Scr, umol/L	144.8 ± 75.7	125.2 ± 53.7	155.7 ± 84.0	0.060
eGFR, mL/min/1.73 m2	49.8 ± 22.9	58.6 ± 25.3	44.6 ± 19.8	0.004
HbA1c,%	7.9 ± 2.3	8.1 ± 2.7	7.5 ± 1.4	0.397
Postdischarge				
Scr, umol/L	145.8 ± 77.0	128.2 ± 54.7	177.4 ± 101.6	0.003
eGFR, mL/min/1.73 m2	47.9 ± 23.2	52.0 ± 23.5	39.8 ± 20.7	0.017
HbA1c,%	7.0 ± 1.6	7.2 ± 1.8	6.5 ± 0.9	0.148

BMI, Body Mass Index; Scr, Serum Creatinine; eGFR, Estimated Glomerular Filtration Rate; HbA1c, Glycated Hemoglobin A1c.

**Table 5:** Odds ratios for CKD rapid progression according to eGFR in patients without AKI during hospitalization.

Variables	Unadjusted OR(95%CI)	P-value	Model 1	P-value	Model 2	P-value	Model 3	P-value
			Adjusted OR(95%CI)		Adjusted OR(95%CI)		Adjusted OR(95%CI)	
eGFR≥60mL/min/1.73 m2	1(Ref)		1(Ref)		1(Ref)		1(Ref)	
eGFR<60mL/min/1.73 m2	3.69 (1.27~10.71)	0.016	3.26 (0.88~12.07)	0.077	2.73 (0.69~10.79)	0.152	7.14 (1.13~45.15)	0.037

Model 1 is adjusted for sex, age, and BMI; Model 2 is adjusted for sex, age, BMI, HTN, and CVD; Model 3 is adjusted for sex, age, BMI, HTN, CVD, and COVID-19 severity.

CKD, Chronic Kidney Disease; CI, Confidence Interval; eGFR, Estimated Glomerular Filtration Rate; OR, Odds Ratio; BMI, Body Mass Index; HTN, Hypertension; CVD, Cardiovascular Disease.

### Incidence of AKI

The incidence of AKI during hospitalization in discharged patients, as subsequently reassessed by renal function, was 35.3% (66/187) (**Supplementary Material S1**). There were no significant differences between the AKI and non-AKI groups in terms of gender, age, or duration of hospitalization. Regarding laboratory parameters, AKI patients had higher lymphocyte counts, D-dimer, fasting glucose, Scr, and NT-proBNP levels compared to non-AKI patients. Additionally, hemoglobin was significantly lower in AKI patients. Patients with AKI received more mechanical ventilation. During the follow-up period, 42 AKI patients survived after discharge, 40% (17/42) of AKI patients had unresolved renal injury, of which 9 patients received long-term maintenance hemodialysis (**Figure 3A**).

## Discussion

Recent studies have shown that the mortality rate of discharged patients with COVID-19 is 4.2% to 6.5% (8, 9). Our research has shown that patients with diabetes and CKD who undergo the acute phase of COVID-19 remain at a high risk of all-cause mortality after discharge, with rates reaching up to 26.7%. This risk was particularly evident in patients with sepsis, and in severe COVID-19 patients receiving mechanical ventilation. Furthermore, our observations suggest that the incidence of COVID-19-related AKI may exceed the rates reported during hospitalization. Additionally, a significant proportion of patients with CKD stages 3 to 5 have been observed to experience rapid deterioration of renal function during the follow-up period.

People aged 65 years and older, those who have not received or have a poor response to the COVID-19 vaccine, and those with multiple comorbidities such as diabetes and CKD are at higher risk for severe COVID-19 (1). Severe COVID-19 can trigger an abnormal immune response characterized by elevated levels of pro-inflammatory and anti-inflammatory cytokines, which may lead to the occurrence of viral sepsis (12). Mechanical ventilation is a critical life-support intervention for patients with sepsis and septic shock. While its use is widespread in the treatment of severe COVID-19, ranging from 12.2% to 40% (9, 10), as our understanding of COVID-19 infection and the advancements in medical care, most patients with severe COVID-19 infection can safely survive the acute phase of the disease and be discharged from the hospital. However, after discharge, patients with sepsis, and patients who have received mechanical ventilation still face a higher risk of death, especially in the first 3 months after discharge, which deserves special attention. Studies have shown that only 50% of sepsis patients fully recover, and about 17% of sepsis survivors have post-sepsis syndrome, which leaves severe persistent physical disability, cognitive impairment, and other long-term effects, and cannot return to the physical state before the onset of sepsis (13). The reasons for the deterioration of health after discharge from sepsis are multifactorial, including the accelerated progression of pre-existing chronic diseases, residual organ damage, and impaired immune function (12). For these patients, the harm of sepsis to them has not ended with infection control and discharge from the hospital after improvement. Mechanical ventilation may be a life-support treatment for patients with severe COVID-19. However, mechanical ventilation is an invasive treatment and lung damage may occur if it is not carefully monitored. Butler MJ et al. (14) reported that among 17,652 patients hospitalized for COVID-19, the post-discharge all-cause mortality was 15.3% (173/1,131) in the mechanical ventilation group and 3.4% (562/16,431) in the non-mechanical ventilation group. It remains a challenge to determine the mechanism of the association between mechanical ventilation and adverse outcomes, whether due to differences in disease severity or consequences of mechanical ventilation itself. Vaccines have played a key role in reducing the severity of COVID-19 and reducing hospitalization rates. However, the effectiveness of vaccines may be reduced in individuals with certain comorbidities, such as obesity, type 2 diabetes, and hypertension (15). Likewise, the effectiveness of vaccines against COVID-19 may decline over time (16). Additionally, as viruses mutate, they may escape the effects of neutralizing antibodies and/or cellular immunity. These are factors that need to be taken into account.

Currently, there are varying reports on the proportion of patients with diabetes and CKD who experience a rapid decline in eGFR each year. A 3-year study found that 14% of patients experienced a significant decline in eGFR (17). Another study found that 61% of patients experienced a rapid decline in eGFR during 6.9 years of follow-up (18). In our study, 35.7% of patients with diabetes and CKD who recovered from COVID-19 experienced a rapid decline in eGFR within one year. Baseline eGFR < 60 mL/min/1.73 m² was identified as the main independent risk factor. Additionally, the progression of renal function may be influenced by concomitant medications, such as antivirals, steroids, and vasopressors, as well as by altered pathophysiological conditions, including acute or chronic kidney injury. Larger-scale, long-term follow-up studies are needed in the future to accurately assess the trajectory of changes in renal function. The higher incidence of hyperglycemia in COVID-19 patients may be related to the release of cytokines induced by COVID-19 infection, which may accelerate metabolic changes by interfering with glucose homeostasis (19, 20). In our study, a downward trend in HbA1c levels was observed after the acute phase of COVID-19 in patients with diabetes and CKD. This may be related to decreased kidney function, reduced insulin secretion, and relatively elevated serum insulin levels. A decrease in HbA1c levels may not represent an improvement in actual blood sugar control, but rather an indirect marker of changes in kidney function.

Although AKI is typically considered a benign, self-limiting, and reversible disease, it is noteworthy that each episode of AKI and increasing severity of AKI increase the risk of progression to CKD, ESRD, and mortality (21, 22). It is now recognized that AKI is a common complication in hospitalized patients with COVID-19. The etiology of COVID-19-associated AKI is likely multifactorial, involving viral sepsis, pneumonia, endothelial damage, hypercoagulability, myocardial dysfunction, drug nephrotoxicity, as well as the effects of general hypoxia and dehydration on renal perfusion (23–25). Patients with diabetes and CKD are more susceptible to AKI than are patients without diabetes or CKD (26), with one study reporting that 35% of patients with COVID-19-associated AKI had underlying CKD stage 3-5 (27). During follow-up, we dynamically monitored Scr values and found that the incidence of COVID-19-related AKI was 35.3%, which exceeded the incidence reported during hospitalization. Although other studies have reported AKI associated with COVID-19 during hospitalization, the patterns of AKI identification in these studies differed (28–31). This may impede the identification of potential AKI and pose a challenge to the reliable detection and staging of AKI. Although most AKI survivors have improved renal function, it is crucial to assess their long-term recovery to ensure appropriate allocation of renal resources (25).

This study has several limitations. Conducted during a particular phase of the COVID-19 pandemic, it faced constraints in data availability, resulting in a relatively small sample size. Due to limitations in the available data, it was not possible to collect information regarding patients’ histories of pulmonary diseases or to record medication use and other potential factors that could exacerbate kidney disease during the follow-up period. Consequently, the study was unable to comprehensively evaluate the impact of pulmonary diseases on the long-term survival of discharged patients or to thoroughly assess the effects of medication interventions. Additionally, the lack of detailed records on the causes of post-discharge mortality restricted further exploration of the mechanisms underlying patient fatalities. A further limitation is that this study was conducted at a single center, with participants primarily consisting of critically ill patients referred from community hospitals. This may have introduced a degree of selection bias, thereby limiting the generalizability of the findings. Given the severity of most patients’ conditions, the results may not fully represent the broader patient population across different healthcare settings, including community hospitals. To ensure the validity of the findings, future research should involve larger, multicenter studies that include a more diverse patient cohort. Such subsequent studies should include detailed analyses of medication use, baseline pulmonary conditions, and other potential factors. Furthermore, the accumulation of long-term follow-up data will facilitate a more comprehensive understanding of the long-term outcomes for patients with diabetes and CKD following COVID-19 infection, offering valuable evidence to guide clinical management.

In conclusion, we observed that patients with diabetes and CKD exhibited higher all-cause mortality and experienced rapid deterioration of kidney function after acute infection with COVID-19 during the one-year follow-up period. This underscores the importance of ongoing longitudinal follow-up to more accurately track the long-term health effects of COVID-19 on patients with diabetes and CKD.

## Data Availability

The original contributions presented in the study are included in the article/**Supplementary Material**. Further inquiries can be directed to the corresponding author.
