# Supplementary material for: COVID-19 in discharged patients with diabetes and chronic kidney disease: one-year follow-up and evaluation
Source: Front Endocrinol (Lausanne). 2025 Feb 4;16:1519993. doi: 10.3389/fendo.2025.1519993 (PMC11832373; doi:10.3389/fendo.2025.1519993)
Supplement: Supplementary file 1 [file Table1.docx]

Supplementary 1 Clinical Characteristics and outcome of Discharged Patients with AKI

| Variables | No AKI | AKI | P-value |
| --- | --- | --- | --- |
| Numebers | (n=121,64.7%) | (n=66,35.3%) |  |
| Male(n,%) | 77 (63.6) | 51 (77.3) | 0.05 |
| Age,years | 69.9 ± 15.7 | 66.4 ± 14.5 | 0.13 |
| COVID-19 severity |  |  | 0.01 |
| Moderate(n,%) | 61 (50.4) | 30 (45.5) |  |
| Severe(n,%) | 31 (25.6) | 8 (12.1) |  |
| Critically severe(n,%) | 29 (24) | 28 (42.4) |  |
| Complications during hospitalization |  |  |  |
| Respiratoryfailure(n,%) | 8 (6.6) | 20 (30.3) | < 0.001 |
| Sepsis(n,%) | 31 (25.6) | 28 (42.4) | 0.01 |
| Myocardial infarction(n,%) | 2 (1.7) | 4 (6.1) | 0.18 |
| Stroke(n,%) | 8 (6.6) | 5 (7.6) | 0.77 |
| Laboratory results within hospital admission |  |  |  |
| Lymphocyte count,×103/μL | 7.6 ± 4.6 | 9.4 ± 5.0 | 0.01 |
| Hemoglobin, g/L | 109.8 ± 25.5 | 98.5 ± 30.3 | 0.008 |
| D-Dimer , ng/mL | 4.7 ± 7.4 | 7.5 ± 9.2 | 0.03 |
| Albumin, g/L | 33.7 ± 5.1 | 31.9 ± 5.8 | 0.03 |
| Total bilirubin,umol/L | 10.7 ± 7.8 | 9.1 ± 7.2 | 0.16 |
| Total cholesterol, mmol/L | 3.6 ± 1.1 | 3.4 ± 1.2 | 0.2 |
| Triglyceride, mmol/L | 2.0 ± 1.7 | 2.1 ± 1.3 | 0.48 |
| Fasting glucose,mmol/L | 10.1 ± 5.2 | 12.8 ± 7.5 | < 0.001 |
| NT-proBNP,pg/mL | 3465.8 ± 5249.6 | 5853.7 ± 7507.4 | 0.02 |
| Scr,umol/L | 165.0 ± 148.0 | 343.6 ± 267.4 | < 0.001 |
| eGFR,mL/min/1.73 m^2^ | 46.7 ± 24.5 | 27.5 ± 23.7 | < 0.001 |
| Uric acid,mmol/L | 347.0 ± 153.9 | 466.0 ± 281.0 | < 0.001 |
| Treatment received during hospital |  |  |  |
| Glucocorticoids(n,%) | 71 (58.7) | 37 (56.1) | 0.72 |
| Antivirals (n,%) | 36 (29.8) | 16 (24.2) | 0.42 |
| Inflammatory factor inhibitors(n,%) | 8 (6.6) | 4 (6.1) | 1 |
| Intravenous immunoglobulin(n,%) | 14 (11.6) | 11 (16.7) | 0.32 |
| Diuretics(n,%) | 93 (81.6) | 55 (87.3) | 0.32 |
| Mechanical ventilation(n,%) | 22 (18.2) | 24 (36.4) | 0.006 |
| Hospital length of stay,days | 14.2 ± 7.7 | 12.1 ± 8.3 | 0.09 |
| Follow up |  |  |  |
| Readmission(n,%) | 11 (10.4) | 14 (31.1) | 0.002 |
| Death(n,%) | 26 (21.5) | 24 (36.4) | 0.02 |

AKI:Acute kidney injury.NT-proBNP:N-terminal pro-B-type natriuretic peptide;Scr:Serum creatinine; eGFR:Estimated glomerular filtration rate.
